# The implementation of the Japanese Dental English core curriculum: active learning based on peer-teaching and learning activities

**DOI:** 10.1186/s12909-019-1675-y

**Published:** 2019-07-10

**Authors:** Omar M. M. Rodis, Rozzano C. Locsin

**Affiliations:** 1 School of Oral Health and Welfare, Faculty of Dentistry, Tokushima University, Tokushima, Japan; 2 Department of Art, Science, and Caring, Institute of Biomedical Sciences, Tokushima University Graduate School, Tokushima, Japan

**Keywords:** Active learning, Peer teaching, Peer learning, Cross-cultural communication, Dental English, Medical English, Student-teacher

## Abstract

**Background:**

Education in Japan and other Asian countries advocates the stereotypical passive learning style where students are limited in their breadth of knowledge dismissing anything not imparted by their teachers. With globalized education, professions are becoming very competitive, embracing student-centeredness compelling them to introduce active learning activities. A study funded by Japan’s Ministry of Education conducted a needs analysis, proposed a solution, and implemented an active learning approach. Since the latter is still new in the Japanese teaching-learning environment, this current study aimed at assessing the willingness of undergraduate students of dental medicine to participate in active learning activities rather than the typical passive-style teaching-learning educational process.

**Methods:**

Three active implementation-learning activities, namely International Group Discussions (IGD), Student-Teacher Experience (STE) and Role Play Activities (RPA) were included in the Dental English course in a classroom setting at a dental school in Japan. Students had to choose between participating in the activity or taking the final examination. Two hundred and three third-year undergraduate dental students participated over a 5-year period from October 2013 to March 2017. For IGD, the researchers assigned students to a topic and grouped them with visiting international exchange students. For STE, researchers gave students teacher-prepared presentation slides on basic dental topics, which they presented in front of their classmates. For RPA, students had to do prepared role-play and impromptu role play. Peer and teacher feedbacks of the activities were given to all students. At the end of the course, the students evaluated the active learning activities and wrote their comments in a free entry survey.

**Results:**

All 203 students participated in the active learning activities confirming the changing learning needs of Japanese students in this dental school. The most common comment was that the class was interesting, fun, an easy-to-understand way to learn dental terms, and a safe way to express themselves in the English language.

**Conclusion:**

The majority of Japanese students preferred the active learning style. The study revealed that students reported greater engagement and better learning with proper guidance and time to prepare for the activities.

**Electronic supplementary material:**

The online version of this article (10.1186/s12909-019-1675-y) contains supplementary material, which is available to authorized users.

## Background

Globalization has become an unstoppable force affecting society, culture, technology, politics, and education worldwide. In the academic field, a majority of universities have started to introduce programs and courses to globalize their faculty, students, programs, facilities, and courses. Professions are now becoming very competitive, embracing student-centeredness and active learning activities. Another effect of globalization is the establishment of a common language, which is English. Unfortunately, most Japanese still lack the ability to express their thoughts in English even after many years of studying. Thus, the Ministry of Education, Culture, Sports, Science and Technology (MEXT) proposed a 5-year ‘Action Plan to Cultivate Japanese with English Abilities’, which aims to improve communicative skills designed to enable Japanese people to share their thoughts and feelings in English in an increasingly global society [1]. Unfortunately, Japan remains to be among the lowest to score in English competency tests and most Japanese are still unable to communicate in English [2]. Some learners with high motivation but with little opportunity to speak English have difficulty in developing the skills of English communication in Japanese classrooms [3]. Moreover, the traditional classroom setting dictates that students should remain quiet, obedient, and not ask questions, reflecting the traits of shyness and humility rooted in their culture [4]. This behavior can create confusion in the interpretation of silence or in being passive in class by the non-Japanese teacher, international students or even among their Japanese peers [5].

Confucianism is the basis of the Japanese educational system, and the core foundation of Japanese teachings reflects the Confucian ideals of filial piety, loyalty to state, submission to authority, and social order [6]. Confucius believed that learning comes from observing and learning from people who provide model behaviors. The main focus of learning is not on questioning, evaluating, and generating knowledge, but on imitating behaviors and character traits of great exemplars. In the Confucian philosophy, asking questions is discouraged because harmony is valued more. This is very evident in most classrooms in Japan. Students never raise their hands to ask questions during the lecture because no one else is raising their hands and doing so will interrupt the flow of learning. In contrast, most Western cultures follow the Socratic philosophy where questioning authorities and finding knowledge within the self by testing personal hypotheses are encouraged. In the West, students actively participate in class by asking questions, by participating in group discussions, or by challenging the teachers. Ideally, active participation could be achieved if Japanese students were exposed to the Confucian educational practices early in life to build character, and then to the Socratic educational practices at a later stage to acquire critical thinking skills and seek the truth [7].

Most national dental schools in Japan accept around 40 students per year. After completing the 6-year dental education program, students become eligible to take the National Dental Practitioner’s Examination and obtain a license. The examination is comprised of 360 basic and clinical multiple-choice type questions. Unfortunately, each year, only one or two questions are in English.

Dental schools in Japan introduced the course “Dental English” in the late 1990s to address a call by the MEXT of Japan, to produce citizens who can function effectively and be competitive in a global society [8]. The course aims to teach English dental terminology and conversational situations commonly used in the field of dentistry [9]. In 2014, a working group approved the core curriculum for Dental English. The working group had been the recipient of a 3-year scientific grant (No. 23531201) (2011–2014) from the Japan Society for the Promotion of Science [10]. In the approved core curriculum, the focus of the Dental English classes was on dental terms and dentist-patient conversation in English. Thus, to implement it, teachers should design active learning styles of classes that can stimulate maximum learning of the topics while giving students the opportunity to be actively involved in the learning and teaching process.

Thus, the aim of this paper was to introduce the implementation of three active learning styles of the Dental English course in the Faculty of Dentistry at Tokushima University. These are the International Group Discussion, for learning cross-cultural communication; the Student-Teacher Experience, for learning dental terminology; and the Role Play Activities, for learning dentist-patient communication.

## Methods

### Description of participants

Two hundred three third year undergraduate dental students of Tokushima University School of Dentistry participated in the International Group Discussions (IGD), Student-Teacher Experience (STE), and the Role-play Activities (RPA). There were 43 students in 2013; 41 students in 2014; 40 students in 2015; 40 students in 2016; and 39 students in 2017. All three activities took place in a classroom setting at Tokushima University Faculty of Dentistry in southwestern Japan over a 5-year period from October 2013 to March 2017.

### The process of active teaching-learning activity

At the beginning of the course, students had to choose between either doing all three activities (IGD, STE, and RPA), or being a member of the audience and take a final written examination. All students received a compilation of handouts for all topics. The class time consisted of 60 min for 16 weeks (Table 1). Weeks 1, 2, and 16 were the only times when the teacher gave a lecture, while Week 3 through Week 15 were for active learning activities. Weeks 3 and 4 were for group discussions with international exchange students concerning ‘*Communication during the Patient’s First Visit.’* Weeks 5 through 9 were for the Student-Teacher Experience in which students acted as teachers to their peers using pre-selected presentation slides. Weeks 10 through 15 were for the Role-Play Activities. There were two sessions for the role-play: the prepared role-play and the impromptu role-play. The role-play was videotaped and saved into a DVD disc. On Week 16, the teacher provided feedback for each role play and a copy of all the videos was given to each student. Moreover, a free entry survey regarding the *three active learning activities* was given to determine whether these active learning activities were effective learning tools for Japanese students.

**Table 1:** Sample dental english schedule for school year 2017–2018

Week	Date	Topic
1	Oct 2	Introduction to the course
2	Oct 10	Etymology
3	Oct 16	Small Group Discussion: What is Communication?
4	Oct 23	International Group Discussion: The patient’s first visit
5	Oct 30	International Group Discussion: Presentation
6	Nov 6	Student-Teacher Experience: Branches of Dentistry 1 (Basic)
7	Nov 13	Student-Teacher Experience: Branches of Dentistry 2 (Clinical)
8	Nov 20	Student-Teacher Experience: The Oral Cavity 1 (Mouth)
9	Nov 27	Student-Teacher Experience: The Oral Cavity 2 (Teeth)
10	Dec 4	Student-Teacher Experience: Oral Diseases
11	Dec 11	Role Play (Prepared role-play + Impromptu role-play)
12	Dec 18	Role Play (Prepared role-play + Impromptu role-play)
13	Jan 15	Role Play (Prepared role-play + Impromptu role-play)
14	Jan 22	Role Play (Prepared role-play + Impromptu role-play)
15	Jan 29	Role Play (Prepared role-play + Impromptu role-play)
16	Feb 19	Teacher’s Feedback

Ethical approval was not required because the study did not involve any experiment and did not ask for sensitive personal information from the students.

### Active teaching-learning activities

#### I. Inter-national group discussion

In the past five years, twenty-four exchange students from the Republic of Indonesia have participated in the international exchange program of the Faculty of Dentistry for a 3-week exchange program. The students participated in the Dental English Class. Students in the class were divided into 4 groups namely: *The Initial Encounter* group, the *Information Gathering* group, the *Communication* group, and the *Closing the Interview* group. The topic was “The patient’s first dental visit”. The international students joined any of the four groups for two weeks. Each group was provided with a large poster-size drawing paper, writing materials and 45 min of class time to discuss their respective topics and make the poster. This encouraged the Japanese students to speak in English so they can relay their thoughts to the international students who would then give a 10-min presentation about the group discussion. The discussions became interesting when students found similarities and differences in their ways of greeting patients (bowing, handshaking, smiling, etc.) during the initial encounter, or when students talk about gestures and choice of words. The groups were free to meet and discuss or practice their presentations before the next class. This provided a good opportunity to get acquainted.

On the day of presentation, the international students presented the poster for 10 min, followed by a 5-min question and answer session with students from the non-presenting groups who were required to ask at least one question. This learning situation enabled the Japanese students to be aware and understand differences in the practice, language, and culture of non-Japanese people without leaving the country. Experts have claimed that engaging with the cultural diversity of students present in the classroom can promote internationalization at home [11]. Moreover, by interacting with classmates and using English to communicate with familiar and unfamiliar people in the group, this two-class activity allowed Japanese students to express their thoughts in the English language and prepared them for the Student-Teacher Experience and Role-playing activities.

#### II. The student-teacher experience

In Japan, dental students become student-dentists or student-clinicians when they are in their 5th and 6th year during the 6-year dentistry course. This allows them to experience real-life scenarios as a dentist in the dental clinic while they are still students. As this practice is familiar to most Japanese students because it is intrinsic in the Japanese culture of an apprentice-master system. A similar approach was integrated into the course to implement the active learning component of the approved Dental English core curriculum. This teaching method received the name ‘Student-Teacher Experience.’

The STE is a peer-learning and peer-teaching activity that allows students to study and present pre-selected presentation slides covering five main topics on basic dentistry to their classmates. The teacher prepares the slides for each topic and gives a copy of the presentation file to each group two weeks before their presentation. The group leader assigns subtopics to each member and they study, practice, and give a lecture during their presentation day. The leader schedules group practices with the teacher and during this time, the teacher can supervise and advise students while other co-members can also have the chance to learn about each other’s topic. Moreover, each member will have the opportunity to actively motivate or encourage each other by giving advice; or to acquire motivation passively by watching their co-members do a good presentation, during their practices. During the actual presentation, peer assessment is done through written free-style commentaries, which the teacher collects, scans, collates, and distributes to each member of the presenting group one week later. Studies have shown that peer-taught lessons bring benefits such as improved motivation, enhanced learning, and authentic communication [12]. The teacher also scores each presenter according to verbal and non-verbal performances.

The STE derives from the active learning concept of student motivation and the adage ‘You learn by doing’. According to Dornyei, there are four stages to motivate students: The first stage is to create motivating condition for learning. This means creating a pleasant and supportive environment in the classroom. The second stage is to introduce initial motivating techniques by creating materials that are relevant for the students. The third stage is to take care to maintain and to protect students’ motivation by offering stimulating activities and fostering self-esteem, self-confidence, and co-operation among students. The fourth stage is to turn evaluation and feedback into positive experiences [13]. These four stages were integrated into the Dental English course as the Student-Teacher Experience. The approach done and the rationale that supports it are presented in Additional file 1.

#### III. The role-play activities

Because role-playing can become a tool to facilitate self and social awareness, it easily gained popularity as a classroom simulation activity in the medical field, especially for doctor-patient relationship and cross-cultural communication competency. The role-play activities were distributed through 5 weeks of the 16-week schedule. Each pair were given one month to decide on their own scenario and role to play, as a Japanese dentist or a non-Japanese patient. As a rule, no scenario should be the same, should be in English, should be at least 8 min, and each pair will have to provide a one-minute statement about the significance of their case scenario at the end of their play. Peer assessment is done by their classmates through comments. Since there are approximately 40 students in the 60-min class, there were around 4 pairs per week for 5 weeks. Each week, two types of role-play activities were scheduled: first, the Prepared Role-Play for the first 45 min, and second, the Impromptu Role-Play, for the remaining 15 min. Details of the Prepared and Impromptu Role Play are presented in Additional file 2.

## Results

All 203 students over a 5-year period participated in the three active learning activities although one group (2 students) did not consent for their role-play to be videotaped. Each student received a DVD containing the full version of all role-plays. For the free entry survey, majority wrote positive comments on the International Group Discussions, Student-Teacher Experience and Role Play Activities.

The 5 most common entries for the International Group Discussions were (verbatim):

1. *Today’s discussion is very fun. The discussion provided a rare opportunity for me to talk with international students.*
2. *I was very fun to play drama with you (international student). Thank you for giving me a precious opportunity.*
3. *Thank you, I’ll practice English and follow your advice. Thank you for a good time. See you again.*
4. *I enjoyed group work together! It was unfortunate that there is only one week left. I want to talk more if there is a chance.*
5. *I was nervous about communication in English, but I enjoyed it. It was a pleasure for you to positively talk to me.*

The 5 most common entries for the Student-Teacher Experience were (verbatim):

1. *This class was very fun and interesting for me and easy to understand. I wasn’t interested in dental terms before, but I started to think that I [should] try to memorize dental terms after I took this class.*
2. *I like your lesson style. I enjoyed every class.*
3. *In the first class, I expected that dental English would be difficult. But this class is very interesting. I think we should do student teacher experience every class.*
4. *I really had fun at this class. All the other class[es] now [are] boring.*
5. *Dental English course was very interesting. I think that learning by Student-Teacher experience and role-play is better than learning by test. Presentation and role-play are good opportunity for us to improve our English proficiency. Thank you.*

The 5 most common entries for the Role-Play Activity were (verbatim):

1. I’m not good at speaking English, so I was afraid to speak English. But Mr. Omar’s English class is very practical and I could meet a lot of foreign country people and make friends. We can actively use English every class and hear foreigners’ English.
2. Thanks to Omar, I met many people. And I decided to go to Indonesia. I’m not good English speaker, so I was avoiding English. But now, I think about touching English more, and I want to meet more people. Thank you, Omar.
3. It’s meaningful for us to do role play in English. I think that the time we treat foreign people is coming soon. This lesson is very interesting. Thank you so much.
4. I enjoyed this class very much. Other class[es] is [are] boring. Other teacher isn’t good at teaching us something. But this class was so interesting! My favorite class is this class!
5. This class is very interesting. I enjoyed the role-play very much. I even want to do it again. Through this class, I started to enjoy speaking English and I want to speak some more.

## Discussion

English has unequivocally become the lingua franca in medicine and its related fields worldwide. Medical and dental universities in EU member states [14, 15] have started revising their medical and dental curriculum by incorporating English while maintaining mastery of national languages. Dental students will have to face stiff competition in the future, not just with dentists in their own countries but those in other countries as well. Teaching a second language in a professional field of study where learners need to master technical terms, doctor-patient communication, and cross-cultural communication, is difficult. Since all of these require the use of English in class for practical and clinical training, Japanese students who are used to learning passively in class, will also have difficulty participating actively and using English in class [16].

The International Group Discussion was a good opportunity for Japanese students to speak English with international students within the safety of their classroom. Since they will discuss on a common topic, there will be less pressure to think about what to talk about. In this activity, dental students from Indonesia will present a summary of their discussion thereby compelling Japanese students to speak English to express their opinions on the topic. Moreover, since English is not the first language for most Indonesians, Japanese students can feel more comfortable as both sides are still learning the language.

The Student-Teacher Experience activity highlighted a new way of mastering dental English terminologies on top of the terminology in the local language, which in this case, was Japanese. Each topic contains its corresponding basic medical and dental terminology and their etymologies which students have to study and master. Being able to know both the common and technical terminology can train students to communicate effectively with non-Japanese patients and colleagues, respectively. Students not only study the terms but use and explain them to their classmates during their presentation as student-teachers. Because they are teaching their peers, they will most likely use easy explanations and simple terms, grammar and syntax. As a group, learning new dental terms in both the local and English language during their practice sessions can also reinforce a better understanding and retention of the term. Although Japan is economically as advanced as western countries, as a country Japan represents a prototypical “conformist” society and inter-dependence-oriented culture, and this could lead to complex psychological and sociological problems [17]. It may still take some time for Japan to fully globalize their educational system while preserving deeply rooted Japanese culture and systems. Nevertheless, globalization is starting to influence the economic, social, political, and even psychological aspects of life in Japan.

The Role Play Activities gave Japanese students the opportunity to experience doctor-patient communication in a familiar classroom setting for both the Prepared and Impromptu Role Play. In the Prepared Role Play, students prepare their own scenario and script. The freedom to choose their own scenario and write their own script allowed them to think of possible clinic situations, the patient’s character, or dental problems, and use simple or easy-to-pronounce words for their scripts. Moreover, deciding, studying and mastering their own case scenario made them confident when they acted in front of their classmates. In the Impromptu Role Play, Japanese students experienced a more realistic doctor-patient communication since the teacher prepared the scenario. Since real international students play the role of the patient, Japanese students will be able to experience the feeling of not knowing the patient, the case, and outcome.

Being able to experience the role-play activities in a safe environment, such as a classroom with their classmates, provides the dental students with an important motivational tool to become competent and empathetic dentists [12–14]. The ability to experience both the feeling of knowing and not knowing will be beneficial in pointing out the importance of English in their profession while motivating them to be globally competent dentists. Although common in most western-style education in which all role-plays should be impromptu role-plays, it must be noted that most Japanese are not used to speaking English in front of other Japanese. Moreover, the Japanese culture of saving face and being a perfectionist also serve as a consideration. Thus, the impromptu role-play immediately following the prepared role-play could be a good solution to reduce anxiety or address concerns of students in making mistakes. Moreover, the use of simulation-based teaching methods is generally considered effective especially with the use of feedback. In the Dental English course, the video recording of role play activities will be primarily for the students’ formative reflective learning when they view their own/classmate’s videos. In fact, some of the participants disclosed that having a copy of the full version of their role-play videos and those of their classmates was very helpful in improving their verbal and non-verbal communication skills because they can watch and review their own performance and those of their classmates, during their free time.

## Limitations and recommendations

A limitation of the study was that the evaluation of the teaching-learning activity made use of free-style entry self-reports, and simple descriptive statistics. Moreover, the present study does not claim to provide a full picture of all Japanese national universities but rather presents only the views of the participants who took part in the survey. While this free-style evaluation provided opportunities for the students to articulate their thoughts in the English language, it was not possible to make some form of plausible prediction because statistical treatments were not included. Nevertheless, as this is the first report on the implementation of the dental English core curriculum, future studies may deal with other concerns, such as the long-term effects of active learning activities using peer teaching and learning activities among various disciplines, professions, and levels of educational curricular placements.

Although the study was limited to Japanese undergraduate students of Dentistry, further studies are also needed to establish the value of active learning, and its application in other countries whose educational system may not be prominently engaged in active learning activities.

## Conclusion

The three active learning activities were able to facilitate a conducive learning environment, introduced motivating techniques, maintained and protected the students’ motivation, and provided effective evaluation and feedback. The activities also increased course content comprehension, built self-esteem and created a sense of community in the classroom through increased student-student and instructor-student interaction. For most students, the International Group Discussion was an activity that motivated them to speak English with international exchange students through a general topic regarding doctor-patient communication; while the Student-Teacher Experience proved to be an effective way of learning dental English terminologies, concepts of oral health and disease; and finally, the Role-Play Activities gave them a chance to experience different scenarios and situations in the clinical setting using English.

This provides evidence that the three activities were manageable and active learning events because students become part of the learning-and-teaching process in a non-threatening, but pleasant, and supportive environment, and because the activities enhances peer-learning and peer-teaching opportunities. The activities also help foster self-esteem, self-confidence, and co-operation among students during practice sessions, and aides the teacher in delivering accurate knowledge which is relevant to the course while being easily comprehensible to the students. Moreover, incorporated activities have become an integral part of the syllabus and have received positive feedback from students in the past five years.

## Additional files

Additional file 1: Principles of the Student-Teacher Experience (STE). (DOCX 8494 kb)

Additional file 2: Principles of the Role Ply Activities (RPA). (DOCX 1478 kb)

## Data Availability

The datasets used and/or analyzed during the current study are available from the corresponding author on reasonable request. Furthermore, all data-generated or analyzed during this study are included in this published article as supplementary information files.

## Abbreviations

DVD: Digital Versatile Disc
IGD: International Group Discussions
MEXT: Ministry of Education, Culture, Sports, Science, and Technology (Japan)
RPA: Role Play Activities
STE: Student-Teacher Experience
