# Supplementary material for: The implementation of the Japanese Dental English core curriculum: active learning based on peer-teaching and learning activities
Source: BMC Med Educ. 2019 Jul 10;19:256. doi: 10.1186/s12909-019-1675-y (PMC6617896; doi:10.1186/s12909-019-1675-y)
Supplement: Supplementary file 1 — Principles of the Student-Teacher Experience (STE). (DOCX 8494 kb) [file 12909_2019_1675_MOESM1_ESM.docx]

**Supplementary File 1**: Principles of the Student-Teacher Experience (STE)

Stage 1. Creating a pleasant and supportive environment: Classroom and Groupings

1.1. Classroom setting and layout

*Approach*: For the dental English course, an ideal classroom should have audio-visual equipment, wireless pin-type microphones, movable chairs and tables, a wide front space. At the dental school of Tokushima University, each year level is assigned an exclusive classroom, which will be their room for the whole school year. Classrooms that have desks fixed on the floor or do not have enough free front space for group presentations or role play activities can prevent teachers from doing some group activities.

*Rationale*: The physical environment of a classroom plays an important part in the ownership students feel about their school and more specifically their class [1]. For example, desks and chairs arranged in neat, orderly rows may make movement easier but may not help create a friendly environment, may not encourage cooperative learning, or build a sense of class community, as with desks arranged in semicircles or clusters [2]. If the course involves small-group discussion or presentation activities, a flexible classroom setup will be ideal. However, if the class has too many students, managing them in an active learning activity may be difficult to manage [3]. Moreover, a good video and audio system will ensure audience attention during lectures and activities. A pin-type wireless microphone is recommended to allow students to practice using their hands for other purposes like using gestures or using the laser pointer. In Japan, hand gestures while speaking are seldom used [4]. By practicing the use of gestures during the Student-Teacher activities, they practice promoting a more encouraging atmosphere [5], a heightened students’ attention [6], the fostering of students’ learning of lesson content” [7,8].

1.2. Groupings

*Approach*: The class is randomly divided into 5 groups in line with the 5 main topics in the syllabus. Since there are approximately 40 students, each group will be composed of 7 to 8 students. Each group will choose a group facilitator or leader who will oversee the basic organization and function of the study group. The leader will facilitate correspondences between the members and the teacher, be the custodian of slides, and schedule group practices.

*Rationale*: Small groupings have been known to create the best peer-learning and teaching environment. The number of slides per topic is about 40 to 50 slides. Therefore, each member will oversee 4 to 6 slides, allowing them to master their presentation and actively take part in giving the lecture to their classmates with confidence. Students will be able to master the subtopics comprehensively and will have enough time to prepare it if there are fewer slides for each member [9].

Stage 2. Introducing motivating techniques/materials: Topics and Slides

2.1. Topics

*Approach*: There are 5 topics on dental terminology allotted for the Student-Teacher experience namely: 1) Branches of Dentistry 1, which introduces the etymology of the names of the basic specializations of dentistry; 2) Branches of Dentistry 2, which introduces the etymology of the names of the clinical specializations of dentistry; 3) The Mouth, which introduces the nomenclature of the parts of the mouth; 4) The Teeth, which introduces the nomenclature of the types and parts of the teeth; and 5) Oral Diseases, which introduces the nomenclature of the common oral diseases on the mouth and teeth. During the student presentations, students in the non-presenting groups write the dental English terminologies on the specified blanks of their hand-outs.

*Rationale*: Most, if not all, medical and dental terminology have their Japanese counterparts in books translated into Japanese, which all students use. Students will be able to take up and master the Japanese terms during last 4 years of dental school. However, since most of these terminologies are derived from Latin or Greek origins, knowing the word root and affixes and word structure can make mastering these terms easier. The strategy ‘to analyze affixes and roots’ engages learners to analyze word structures [10]. In the said study, students were asked to rank strategies like verbal repetition, written repetition, use of dictionary, etc., that they used most and least frequently. The study concluded that written repetition had the highest frequency. Thus, writing the new term on the handouts can be effective in vocabulary retention. Furthermore, the slides contain both the new English term and the familiar Japanese term, making retention of the English terminology much easier.

2.2 Slides

*Approach*: The slides are prepared by the teacher in advance using a commercially-available presentation software before the first day of classes starts, saved into a USB flash drive, and distributed to the leader of the first group at least two weeks before their group gives the lecture. The leader gives a copy to each member and they will decide which subtopic to have for themselves. Once each of the member has decided on their subtopic, they can copy the slides for their part, study it, and write their own script based on their own understanding of the contents of the slides. Meanwhile, every week henceforth, the teacher gives a flash drive with the slides for the next topic to the leader of the next group. This will give each group exactly two weeks to prepare for their lecture. If students want to modify their slides for ease in understanding, they may do so with the permission from the teacher. For example, slides regarding Branches of Dentistry group will have dental terminologies including definition, word root, Japanese translations, and other pertinent information such as name and head of the department. The non-presenting groups will receive the printed version of the presentation slide with blanks which they must fill out during the lecture. These handouts will be distributed to students of the non-presenting groups on the day of the lecture. Members of the presenting group will have handouts that already have the answers to the blanks.

*Rationale*: It is important that the slides should be prepared by the teacher and not left to the students to make because it will ensure that all important information for the subtopics will be covered. With the permission from the teacher, the students may modify or add/delete slides if they feel that some of the slides prepared by the teacher is difficult to understand. For active learning to be effective, learners must have the opportunity to use available resources, make decisions, and be creative. Because they make their own scripts by studying and researching about their subtopics by themselves, they are learning by themselves and when they make the presentation, they are teaching their classmates [11]. This makes retention of knowledge easier. Moreover, by writing their own script, it is assured that they will use simple words, which will be easy for their classmates to understand. As for the audience, they are forced to pay attention to the lecture because they must fill in new terminologies on their printouts.

Stage 3. Offering stimulating activities: Rehearsal and Presentation

3.1. Rehearsals

*Approach*: As a rule, groups cannot make their presentation in front of their classmates without practicing. Group practices must be done at least three times with the teacher. Individual practices may be done as often as possible. Schedules for group practices will be decided by the leaders after consulting the group members. Practices with the teacher may be done during lunch break or during the group’s free time with all members of the group present. The first practice is usually for finalizing the slides, correcting the scripts, and adjusting individual and group timings. Succeeding practices will be for mastering content, delivery and presentation techniques without reading the scripts. During their practices, they can ask someone in their group to answer questions that they intend to ask to their classmates during the actual presentation.

*Rationale*: It must be noted that this experience of giving a lecture or presentation in English will most likely be their first time. Thus, the more practices they do, the more they get the chance to master their topics, build self-confidence, and overcome shyness. Each group may practice as many times as possible but they must practice with the teacher at least three times. The practices will be important because each member can be guided accordingly by the teacher [12]. The guidance from the teacher will also ensure that each member will be explaining their content thoroughly and accurately. Verbalizing their ideas of the slide helps reinforce the concept and improves knowledge retention of their own topic. Group practice offers the opportunity to get support from their group members through suggestions or advice on how to explain their topic in a simpler way or using an easy-to-understand English term [13]. Moreover, watching their groupmates make a good presentation can motivate and encourage them to practice more and strive harder to also do a good presentation, compared to when they are practicing alone. Asking questions during the practice can allow their groupmates to be also familiar with one’s own subtopic thereby allowing the whole group to master their group topic. Since subtopics are interrelated with each other, for example, enamel, dentin, and cementum, comprehension of the general topic is enhanced. Moreover, most Japanese are not familiar with the basic Western-style presentation techniques, like making gestures or establishing eye contact. Thus, practicing will also be an opportunity for them to learn these techniques. Because communication skill building requires risk-taking on the part of the learner, the development of a safe learning environment by the educator is critically important [14].

3.2. Presentation

*Approach*: Since Dental English is a 60-minute class, each group presentation is allotted 45 minutes and reading of scripts is not allowed. The presenters can use tooth models, other visual aids, or do role plays to explain their topics. They can also ask questions to their classmates as part of their lecture. At the end of their presentation, at least eight students from the non-presenting groups must ask one question to each of the presenters. By the end of the course, all students will have had the chance to ask a question to their classmates. Questions or topics that are left unaddressed will be clarified by the teacher. A general feedback to the presenting group is also given by the teacher at the end of the class.

*Rationale*: Each group is allowed two weeks to prepare because it is important to give each member the chance to master their own part of the presentation and help reduce shyness and anxiety in speaking English in front of their classmates. For most Japanese, making mistakes and feeling humiliated in front of peers can be detrimental in building self-confidence. Culture also plays a role in determining people’s responses to humiliation [15]. In Japan, saving face is valued above all else [16]. The teacher should make sure everyone in the group has had enough practice before making the actual presentation. This can be managed during the group practices by guiding and advising slow learners. Since they wrote their own scripts based on their understanding of the slides, remembering it will be easier. Involving their classmates with their own topic by asking questions can also promote same-level interaction with each other, which may reduce anxiety and increase mutual interest on the topic.

Stage 4. Turning evaluation into positive experiences: Feedback and Documentation

4.1 Feedback

*Approach*: Feedback by peers is done by those in the non-presenting groups through comments and giving a score on the Student-Teacher Shuttle Cards [17] to each member of the presenting group (Fig. 1). The comments are scanned, cropped, then collated into a one-page feedback sheet, and given back to the group members (Fig. 2). Prior to the start of the presentations, students will have to practice giving comments and scoring through a short video on doctor-patient communication while comparing their scores with their peers and the teacher. Following the Ladder of Feedback [18], peers should ask questions to clarify and gather relevant information before giving the feedback. Then, they can comment on the strengths of the presentation or mention the points that they value. After which, the peers can give comments on points that may be of concern and then provide suggestions on how those concerns can be improved to help the student being assessed address those concerns. Scoring by the teacher is done using a 10-level score, where 1 is the lowest score and 10 is the highest, for verbal and non-verbal communication and for knowledge of topic. Thus, each member will have a total of 30 points for their presentation. Indicators for verbal communication include clarity and speed; non-verbal communication include eye contact and gesturing or use of laser pointer or facial expressions; and knowledge of topic includes mastery (not memorizing/not reading script), and self-confidence (delivery, asking/answering questions). Furthermore, the teacher provides constructive criticisms to the group at the end of the presentation, by pointing out both the good and bad points, while providing ways or techniques to overcome or correct mistakes.

*Rationale*: Trainees crave genuine feedback based on direct observation [19]. Thus, teachers can have the opportunity to provide this during and at the close of a teaching encounter in front of the group and, if necessary, privately, later [20]. The value of feedback has been known to enhance student learning [21]. The role of peer assessment has also been important in this process as having the potential to improve student learning, particularly in the context of formative assessment [22,23].

4.2 Documentation

*Approach*: All presentations are video-taped. This is announced at the beginning of the course and a week before the activity starts. The students are provided an explanation on the purpose of recording their presentations. If they do not want their role-play to be recorded, they are free to refuse without any consequences. At the end of the course, a video compilation of all the full presentations are distributed to all students in the form of an MP4 file saved on a DVD. Moreover, a video digest, a 10-minute video clip compilation, will also be made for educational purposes and shown to the next batch of students.

*Rationale*: A copy of the videos will be given to each student. This is intended to enhance learning, self-assessment and peer-assessment in the future. Since the Dental English course is offered only for one semester out of the twelve semesters of dental student life, this will be their only chance to make presentations in English. More recently, there is strong evidence that video reporting can inspire and engage students when incorporated into student-centered learning activities by increasing student motivation, enhancing learning experience, give students higher marks, develop the potential for deeper learning of the subject, develop of learner autonomy, and can enhance team work and communication skills [24]. Students will be able to see their own performance and compare it with their peers. Self-assessments can help a student and teachers acknowledge what is wrong and how they can correct it [25]. Moreover, both peer teaching and learning also helps boost understanding among students throughout the course.

**REFERENCES (21-45)**

1. Bucholz JL and Sheer JL. Creating a Warm and Inclusive Classroom Environment: Planning for All Children to Feel Welcome. Electronic Journal for Inclusive Education. 2009;2(4).
2. Patton JE, Snell J, Knight W, et al. A survey study of elementary classroom seating designs. ERIC Document. 2001;454-194.
3. Exeter DJ, Ameratunga S and Ratima M. Student engagement in very large classes: the teachers’ perspective’. Studies in Higher Education. 2010;35(7):761-775.
4. Nishiyama K. Doing business with Japan: Successful strategies for intercultural communication’. Honolulu, HI: University of Hawaii Press; 2000.
5. Stam G and McCafferty SG. Gesture studies and second language acquisition: a review’. In S. G. Mccafferty & G. Stam (Eds.), Gesture: Second Language Acquisition and Classroom Research; 2008. p. 3-24. Routledge: London.
6. McCafferty SG. Gesture and creating zones of proximal development for second language learning. Modern Language Journal. 2002;86:192-203.
7. Quinlisk CC. Nonverbal communication, gesture, and second language classrooms: a review. In S.G. McCafferty & G. Stam (Eds.), Gesture: Second Language Acquisition and Classroom Research; 2008. p. 25-44. Routledge, London.
8. Alibali MW and Nathan MJ. Teacher’s gestures as a means of scaffolding students’ understanding: Evidence from an early algebra lesson. In R. Goldman, R. Pea, B. Barron, & S: J. Derry (Eds.) Video Research in the Learning Sciences; 2007. p. 349-365. Mah Wah, NJ: Erlbaum.
9. Villiers M. The value of small group learning: an evaluation of an innovative CPD program for primary care medical practitioners. Medical Education. 2003;37:815-821.
10. Yang MN. Nursing Pre-professionals' Medical Terminology Learning Strategies’ Asian EFL Journal. 2005;7(1):9.
11. Fukuda T and Yoshida H. Time is of the essence: factors encouraging out-of-class study time. ELT Journal. 2013;67(1):31-40.
12. Mennim P. Rehearsed oral L2 output and reactive focus on form. ELT Journal. 2003;57(2):130-138.
13. Secomb J. A systematic review of peer teaching and learning in clinical education. J Clin Nurs. 2008;17:703-716.
14. Bransford JD and Brown AL. How People Learn: Brain, Mind, Experience, and School. Washington DC: The National Academies Press; 2000.
15. Otten M and Jonas KJ. Humiliation as an intense emotional experience: Evidence from the electro-encephalogram. Social Neuroscience. 2014;9(1):23-35.
16. Hasada R. Cultural scripts: Glimpses into the Japanese emotion world. In C. Goddard (Ed.), Ethnopragmatics. Understanding discourse in cultural context. 171-198. Berlin: Mouton de Gruyter; 2006.
17. Rodis O, Kariya N, Matsumura S, et al. The Student-Teacher shuttle card for Japanese dental students taking a dental English course. TESOL. 2010;2(1):73-90.
18. Perkins D. King Arthur’s Round Table: How Collaborative Conversations Create Smart Organizations. Hoboken, NJ: John Wiley & Sons; 2003.
19. Kurtz S and Silverman J. Teaching and learning communication skills in medicine. Oxford: Radcliff; 2005.
20. Jackson V and Back A. Teaching Communication Skills Using Role-Play: An Experience-Based Guide for Educators. J Palliat Med. 2011;1(6):775-780.
21. Orsmond P and Merry S. Implementation of a formative assessment model incorporating peer and self-assessment. Innovations in Education and Training International. 2004;41(3):273-290.
22. van den Berg I. and Admiral W. Student peer assessment in higher education: Analysis of written and oral peer feedback. Teaching in Higher Education. 2006a;2:135-147.
23. Cartney P and Rouse A. 2006. The emotional impact of working in small groups: High- lighting the influence on student progression and retention. Teaching in Higher Education. 2006;11(1): 79-91.
24. Willmot P. and Bramhall M. Using digital video reporting to inspire and engage students. http://www.raeng.org.uk/education/hestem/heip/pdf/Using_digital_video_reporting.pdf. Accessed 25 Oct 2017.
25. Skovholt, K. Anatomy of a teacher-student feedback encounter. Teaching and Teacher Education. 2018;69:142e153.

Figure Titles:

Figure 1 Cropped and collated entries for peer feedback

Figure 2 Teacher’s score sheet for the Student-Teacher Experience
