# Supplementary material for: The implementation of the Japanese Dental English core curriculum: active learning based on peer-teaching and learning activities
Source: BMC Med Educ. 2019 Jul 10;19:256. doi: 10.1186/s12909-019-1675-y (PMC6617896; doi:10.1186/s12909-019-1675-y)
Supplement: Supplementary file 2 — Principles of the Role Ply Activities (RPA). (DOCX 1478 kb) [file 12909_2019_1675_MOESM2_ESM.docx]

**Supplementary File 2**: Principles of the Role Ply Activities (RPA)

Part 1. The Prepared Role-Play

*Approach*

Role-playing have been used to train medical and paramedical students for clinical and communication skills in the medical field. There are many ways of organizing role-play activities including peer role-playing or the use of simulated and standardized patients [1,2]. In the prepared role-play activity, students are given the freedom to choose their scenario and respective roles two weeks before their scheduled role play. The time allotted to plan, practice and prepare can help students build confidence. This is important especially for Japanese students, who are not used to public speaking, debate, and speaking English in front of their classmates.

With the time given to prepare their setup, materials, checking the sound system, and including the total role-play time, each pair will have about 12 minutes in total. It is expected that all 4 role-plays will be finished 45 minutes past the start time. Students were paired with a partner according to their seating arrangement and play the role of a Japanese dentist and a non-Japanese patient/dentist. Both students decide by themselves on which roles to take and the scenario to play. Scenarios can be fictional or based on actual experience and must include the basic elements of medical/dental interviewing like opening the interview, data gathering, verbal/nonverbal communication, and closing the interview (Fig. 3). Students write the script of their scenario and once they finish it, they have to send it to the teacher for review before they can use it for their role play. They must rehearse with the teacher at least once. The rules for the role players were to: speak English, not read scripts, perform for at least 8 minutes, choose an original, challenging and engaging case scenario, and to present a 2-minute introduction and/or summary of the practical implication/s of the of their role play in Japanese.

*Sample prepared role play scenarios*:

1. Impacted 3rd molar of a medically-compromised male patient (Batch 2013).
2. Pregnancy gingivitis and tetracycline antibiotics (Batch 2014).
3. Difficult to manage hearing-impaired elderly female patient (Batch 2015).
4. Metal denture allergy during a flight with a pilot-dentist (Batch 2016).
5. White coat phobia of a father-daughter pair (Batch 2017).

Students in the non-presenting groups must write their comments to the person playing the role of the dentist, on their shuttle card. For peer feedback, the comments their classmates wrote are scanned, cropped, and collated as one feedback sheet, then distributed back to the respective role players in the following week (Fig. 4). For teacher feedback, the student playing the role of the dentist is scored through a score sheet containing the 4 elements of medical/dental interviewing (Fig. 5). Students were reminded of the criteria for grading at the beginning of each session. These scores are added to the Student-Teacher Experience grade and makes up a major part of their final grade.

*Rationale*

The two-week preparation time will give students enough time to decide on which scenario to play and practice. They can also ask advice or borrow the videos of their seniors. However, as a rule, no scenario should be the same as those in any of the previous years. Students can then study the scenario, look for interesting cases, plan the correct or incorrect diagnosis, treatment, and prognosis of their case, and check patient behavior and compliance, by themselves [3]. Studying their own case scenario makes them confident when they act in front of their classmates. Because the teacher reviews their script for accuracy of information, parts of the dialogue that needs clarification is discussed with the students. Once the script is finalized, they can start rehearsing by themselves and then with the teacher. Rehearsals with the teacher should be done at least once.

Role players can also get a compiled version of the feedback from their classmates, which is a collated version of feedback from the classmates. A common comment or opinion can help role players realize their major mistakes or good points through the perspective of the audience. Moreover, they can watch a video of their role play to confirm the good and bad points of their role play at the end of the course or at a future time [4].

Part 2. The Impromptu Role-Play

*Approach*

The last 15 minutes of the 60-minute class will be for the Impromptu Role Play. This time, the case scenario is decided by the teacher who prepares the scenario and slides related to it. These slides could be an x-ray or intra-oral photo, dental materials, or treatment plans, and will be on stand-by in case it is needed. One of the 8 students performing the role-play for the specific day will play the role of the dentist. The activity is voluntary but if there will be no volunteers, the role player will be drawn by lots. Depending on the scenario, other role players may be drawn by lots to act as assistants or other health care professionals. When the role-player is ready, the teacher projects the scenario onto the screen for everyone to see and the activity starts. There is no time limit for the activity. However, the role player must perform the 4 elements of medical/dental interviewing. Instead of the student-audience giving feedback, it is the teacher who points out the good and bad points of the role-play after the activity ends.

*Sample impromptu role play scenarios*:

1. Consultation concerning pain in the back teeth area of a Finnish tourist in Japan.
2. Dental needle phobia leading to syncope of a Chilean couple visiting Japan.
3. Bleeding gums and tooth ache of a pregnant Indonesian patient who is a Muslim.
4. Nursing caries and dental fear in an Indian child patient with her inquisitive mother.
5. Shallow dental caries of an elderly Filipino patient with mild dementia who wants the tooth extracted.

*Rationale*

The Impromptu Role Play is an important part of the role play activities because it can represent an actual or typical first-visit scenario in the clinic where dentists and patients do not know each other. In the real case scenario, dentists will not know what kind of patients are coming and what oral conditions they have. The students playing the role of the dentist are drawn by lots thereby giving them the opportunity to experience the feeling of being uncertain of the unknown or to be in the dentist’s shoes. However, being able to experience it in a safe environment such as a classroom with classmates is considered an important motivational tool for dental students to become a competent and empathetic dentist [5]. Since they have just done their prepared role-play, it is presumed that they are still familiar with the elements of a good medical/dental interview, flow of the interview, and the commonly-used terms. Moreover, remembering important information under pressure will be beneficial for the long-term retention of an experience and a newly acquired knowledge.

**REFERENCES (50-54)**

1. Luttenberger K, Graessel E, Simon C, and Donath C. From board to bedside – training the communication competences of medical students with role plays. BMC Med Educ. 2014;14:135.
2. Bosse HM, Schultz JH, Nickel M, Lutz T, Möltner A, Jünger J, Huwendiek S, Nikendei C. The effect of using standardized patient or peer role play on ratings of undergraduate communication training: a randomized controlled trial. Patient Educ Couns. 2012;87: 300-306.
3. Bosse HM, Nickel M, Huwendiek S, Junger J, Schultz JH, Nikendei C. Peer role-play and standardised patients in communication training: a comparative study on the student perspective on acceptability, realism, and perceived effect. BMC Med Educ. 2010;10:27.
4. Nisbet G, Jorm C, Roberts C. Content validation of an interprofessional learning video peer assessment tool. BMC Med Educ. 2017;17:258.
5. Rotzoll D, Wienhold R, Weigel Anni, Wolf R. Present situation of and future outlook for undergraduate English for medical purposes education in Germany. J Med Eng Educ. 2014;13(3):47-54.

Figure 3 Four elements necessary for effective medical/dental interviewing

Figure 4 Cropped and collated entries for the role-play peer feedback

Figure 5 Teacher’s score sheet for the role-play activity
